# Mechanistic Insights into the Antimicrobial Actions of Metallic Nanoparticles and Their Implications for Multidrug Resistance

**DOI:** 10.3390/ijms20102468

**Published:** 2019-05-18

**Authors:** Sibhghatulla Shaikh, Nazia Nazam, Syed Mohd Danish Rizvi, Khurshid Ahmad, Mohammad Hassan Baig, Eun Ju Lee, Inho Choi

**Affiliations:** 1 Department of Medical Biotechnology, Yeungnam University, Gyeongsan 38541, Korea; sibhghat.88@gmail.com (S.S.); ahmadkhursheed2008@gmail.com (K.A.); mohdhassanbaig@gmail.com (M.H.B.); gorapadoc0315@hanmail.net (E.J.L.); 2 Amity Institute of Molecular Medicine and Stem Cell Research, Amity University, Noida 201313, India; nazianazam@gmail.com; 3 Department of Pharmaceutics, University of Hail, Hail 2440, Saudi Arabia; syedrizvi10@yahoo.com

**Keywords:** nanomaterials, antimicrobials agents, drug resistance, physico-chemical property, superoxide radicals

## Abstract

Multiple drug-resistant bacteria are a severe and growing public health concern. Because relatively few antibiotics have been approved over recent years and because of the inability of existing antibiotics to combat bacterial infections fully, demand for unconventional biocides is intense. Metallic nanoparticles (NPs) offer a novel potential means of fighting bacteria. Although metallic NPs exert their effects through membrane protein damage, superoxide radicals and the generation of ions that interfere with the cell granules leading to the formation of condensed particles, their antimicrobial potential, and mechanisms of action are still debated. This article discusses the action of metallic NPs as antibacterial agents, their mechanism of action, and their effect on bacterial drug resistance. Based on encouraging data about the antibacterial effects of NP/antibiotic combinations, we propose that this concept be thoroughly researched to identify means of combating drug-resistant bacteria.

## 1. Introduction

Antibiotic-resistant bacteria, especially multidrug-resistant strains, have emerged over recent years [1,2,3]. Antibiotics are preferred for the treatment of infections caused by these bacteria, because of the results achieved and their cost-effectivenesses. Although the evolution of multidrug-resistant strains presents an important clinical problem [1,2,4], currently “super bacteria”, which arose as a result of antibiotic abuse, are attracting more attention as they are resistant to almost all known antibiotics. The level of antibiotic resistance they display is credited to the presence of a super-resistance gene named ‘New Delhi metallo-beta-lactamase 1’ [5].

Bacterial targets of most antibiotics in present use are related to (1) cell wall synthesis, (2) DNA replication, and (3) translational machinery. Unfortunately, bacteria are skilled at developing resistance irrespective of target functionalities. The various resistance mechanisms used include (1) expressions of enzymes capable of altering or degrading antimicrobial agents such as aminoglycosides and beta-lactamases [6]; (2) cell wall alterations, enzymatic drug cleavage or modification, ribosomal mutations, 16S rRNA methylation, and alterations in the β-subunit of RNA polymerase and carbapenemases that provide resistance against different antibiotics [7]; and (3) the expressions of drug-specific efflux pumps [8]. 

Nanotechnology is an emerging field with enormous scope, and nanomedicine provides an excellent platform for overcoming the problem of drug resistance [9]. In addition to their role in combating antibiotic resistance, nanoparticles (NPs) have the potential to inhibit advanced glycation end-products formation [10] and to be used in anticancer therapies [11]. Nanoformulations containing antimicrobial agents also allow dosage reductions and enhance antimicrobial activities. Conjugating antimicrobial agents and NPs also improve abilities to kill microbial pathogens that have developed antimicrobial resistance. Additionally, antibiotic-conjugated NPs enhance antibiotic concentrations in sites of bacterium–antimicrobial interaction and aid the binding of antimicrobial agents to bacteria [12]. 

## 2. Antibacterial Mechanisms of NPs

Bacterial colonization on solid surfaces leads to biofilm formation and is a major cause of nosocomial infections. Bacterial flagellum synthesis is inhibited after surface attachment, and subsequent rapid bacterial growth results in biofilm formation [13]. In addition, microbial aggregates in biofilms produce a blockade that resists antimicrobial agents, and the bacterial communities of biofilms evade the immune system by producing superantigens [14]. The secretion of extracellular polymeric substances also causes permanent attachment of bacteria to surfaces.

A surge in the usage of NPs in the medical field has opened opportunities for extensive research on their antibacterial mechanisms [15]. Metal NPs potently alter the metabolic activity of bacteria as evidenced by Chatzimitakos and Stalikas [16], and could be of considerable use for the treatment of bacterial diseases. In addition, Ag containing NPs can enter biofilms and prevent biofilm development by suppressing gene expression [17].

The extremely small dimensions of NPs are useful for accomplishing antimicrobial actions and fighting intracellular bacteria [14]. In general, small sized silver (5, 9, 10, 12, and 13.5 nm), gold (8.4 nm), zinc-oxide (12 nm), and titanium-oxide (12 and 17 nm) NPs have high antimicrobial activities [18]. Since these particles act only when in contact with bacterial cell walls, various means of promoting NP-bacterial contact, such as electrostatic attraction [19], Vander Waals forces [20], and receptor–ligand [21] and hydrophobic interactions [22], have been studied. After making contact, NPs can cross microbe membranes, interfere with metabolic pathways, and induce changes in membrane shape and function. Once inside cells, NPs interact with the microbial cellular machinery to inhibit enzymes, deactivate proteins, induce oxidative stress and electrolyte imbalance, and modify gene expression levels [23]. In this part of the review, we highlight some of the more important antibacterial mechanisms of NPs.

### 2.1. Oxidative Stress

Reactive oxygen species (ROS)-induced oxidative stress is a vital aspect of the actions of NPs against bacteria. The four ROS types are the superoxide radical (O^−^), the hydroxyl radical (·OH), hydrogen peroxide (H_2_O_2_), and singlet oxygen (O_2_). O^−^ and H_2_O_2_ are from short-term stress reactions and can be reduced by endogenous antioxidants such as superoxide and catalases. Singlet oxygen (O_2_) accounts for much of the physiological damage caused by ROS [24]. Under normal conditions, equilibrium is maintained between the generation and clearance of ROS in bacterial cells, but when ROS production is excessive, the intracellular redox state is altered and supports oxidation [25].

Oxidative stress is a key contributor in altering the bacterial membrane permeability, and thus can damage cell membranes [26]. Nano silver ions activate oxygen and produce reactive oxygen ions and hydroxyl radicals, which can hinder bacterial proliferation or destroy bacterial cells [27]. Similarly, Al_2_O_3_ NPs can cross, interact, and eventually destroy bacterial membranes by inducing oxidative stress within bacterial cells [28]. 

### 2.2. Dissolved Metal Ions

Metal ions are gradually released from metal oxides in aqueous medium and are subsequently absorbed through cell membranes, which in turn leads to direct interactions with the functional groups of proteins and nucleic acids (e.g., mercapto (–SH), amino (–NH), and carboxyl (–COOH) groups). These interactions have wide-ranging effects, which include cell structural changes and aberrant enzyme activities, which perturb normal physiological processes [29]. Palladium nanolayers (ranging in size from 0.4 to 22.4 nm) and silver nanowires (20 nm) prepared on polyethylene naphthalate were observed to have antibacterial effects, which were attributed to the release of palladium and silver ions to solution [30,31]. Conversely, only weak antibacterial activity was observed when metal oxide suspensions were added to bacterial cultures, which suggests metal ion dissolution is not responsible for the antibacterial effects of metal oxide NPs [32]. Furthermore, it was observed that media containing metal oxide NPs eluted from dental composite resins did not have antibacterial activity, which suggests a threshold NP concentration must be achieved [33]. 

### 2.3. Non-Oxidative Mechanisms

Nanomaterial-based techniques such as electron spin resonance spectroscopy, liquid chromatography-mass spectrometry, Fourier transform infrared analysis, proteomics tools, transmission electron microscopy, and flat cultivation have been used to assess the antimicrobial activities of MgO. Under UV and natural light, three types of MgO NPs have been confirmed to have antibacterial activity against *Escherichia coli*. However, these antimicrobial activities of NPs are unrelated to membrane lipid peroxidation due to oxidative stress because (1) when microbial cell membranes break, surface pores are observable, MgO NPs are not found in cells, and no intracellular magnesium ion excess is detected spectroscopically. These observations indicate that MgO NPs damage cell membranes, possible due to a combination of NP to membrane attachment, the effects of pH changes, magnesium ion release, and UV illumination; (2) only small amounts of intracellular ROS are present; (3) treatment with MgO NPs does not significantly change phosphatidylethanolamine or lipopolysaccharide (LPS) levels in cell walls, indicating MgO NPs do not induce lipid peroxidation. Furthermore, levels of ROS-associated intracellular proteins do not change, but several important metabolic processes associated with proteins involved in amino acid, carbohydrate, and nucleotide metabolism are markedly reduced [34].

### 2.4. Types of NPs and Antimicrobial Potentials

**Figure 1:**
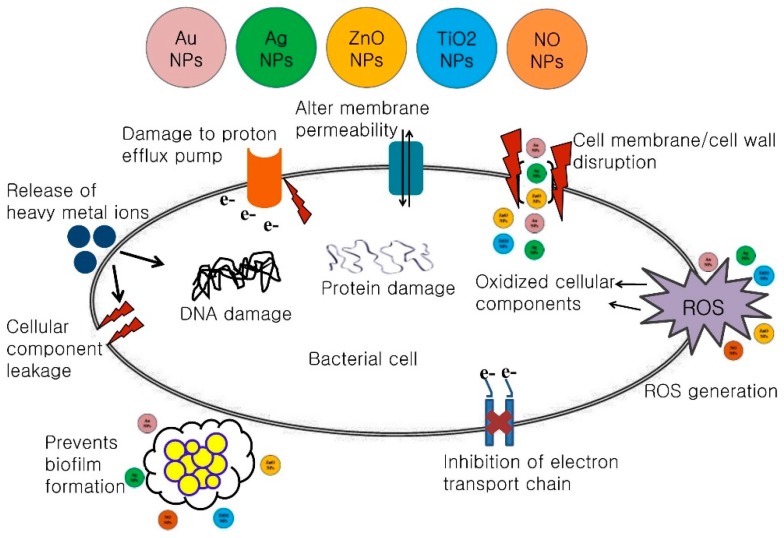
Schematic representations of the antimicrobial mechanisms of various nanoparticles (NPs).

A vast range of NPs engineered from various nanomaterials have been synthesized [35]. The majority of nanomaterials described in recent studies have antibacterial activity attributable to at least one of the following mechanisms: inhibition of cell wall/membrane synthesis, disruption of energy transduction, production of toxic ROS, photocatalysis, enzyme inhibition, and reduced DNA production [36]. Some well-documented antimicrobial nanomaterials are described in Table 1 and their probable antibacterial mechanisms are detailed in Figure 1.

### 2.5. Gold NPs

Gold nanoparticles (AuNPs) represent a revolution in drug delivery, and are considered safe and non-toxic antimicrobial agents. Rai and colleagues studied the mechanism responsible for the antimicrobial action of cefaclor conjugated AuNPs, and reported observed bactericidal activity was the result of synergism between cefaclor and AuNPs. Upon interaction with the outer peptidoglycan layer, cefaclor enhanced membrane porosity and AuNPs also created holes in cell walls. Thus, it was suggested cefaclor-capped AuNPs penetrate bacterial membranes and interact with DNA. In addition, they suggested interaction between AuNPs and DNA hindered DNA unwinding and transcription [43,44]. Furthermore, treatment with cefaclor conjugated AuNPs resulted in high cefaclor concentrations in bacteria [44].

**Figure 2:**
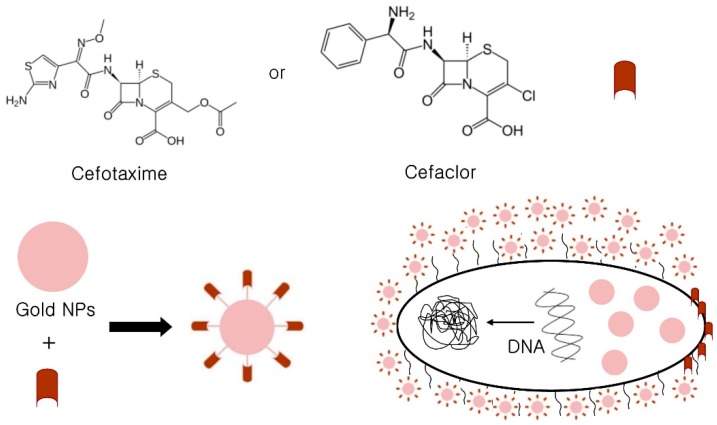
Proposed antibacterial mechanisms of antibiotic-conjugated AuNPs. AuNPs act as antibiotic carriers and facilitate access to bacterial cell walls. Cefaclor or cefotaxime damage cell walls and enable AuNP entry, and AuNPs then prevent DNA from unwinding.

Shaikh and co-workers observed a similar mechanism for cefotaxime-capped AuNPs against drug-resistant microbial pathogens that generate extended spectrum beta-lactamase (antibiotic-degrading enzymes) [45]. It was postulated high cefotaxime concentrations were deposited in bacterial cells by AuNPs and that this might inhibit the cell wall before being degraded by bacterial beta lactamase enzyme. In addition, AuNPs also damaged bacterial DNA. Figure 2 illustrates and summarizes the antibacterial mechanisms of antibiotic-conjugated AuNPs. However, further research is needed to establish more accurately the mechanisms responsible for the antimicrobial actions of AuNPs against different bacterial pathogens. 

Mohamed and colleagues described the antimicrobial effects of AuNPs (25 nm sized prepared by co-precipitation) against *Corynebacterium pseudotuberculosis*. Three doses of AuNPs (50, 100, or 200 μg/mL) were tested to determine minimum inhibitory concentration (MIC) and microbial growth rates. AuNPs exert their antibacterial activities via ROS generation, and thus increase oxidative stress in bacterial cells, which leads to vacuole formation (a marker of effective antibacterial activity). The antimicrobial activities of these NPs were also increased by laser light. The MICs of AuNPs and of combined AuNP/laser therapy were 200 μg/mL and 100 μg/mL, respectively, showing combinations of AuNPs and laser exposure offer a possible means of treating *C. pseudotuberculosis* infections [46].

AuNPs and methylene blue were used to prepare light-activated antibacterial polymers using a simple swell-encapsulation-shrink technique. The polymers synthesized potently reduced bacterial survival. AuNPs increased the hydrophobic properties of polymers and enhanced bactericidal activity by enhancing the generations of ROS other than singlet oxygen [47]. Furthermore, the antimicrobial action of chitosan-conjugated AuNPs loaded with ampicillin was reported to be twice that of ampicillin alone against *E. coli*, *S. aureus*, and *K. mobilis* [37]. Similarly, amino-substituted pyrimidines lacking antimicrobial activity exhibited antibacterial activity against multi-drug-resistant *E. coli* and *Pseudomonas aeruginosa* isolates when conjugated with AuNPs in the absence of an energy source such as visible light [48]. The mechanism responsible was explained as follows: pyrimidine-conjugated AuNPs sequester magnesium or calcium ions, causing cell membrane disruption and leakage of cellular components, and internalized NPs inhibit the synthesis of DNA and proteins. Furthermore, amino-substituted pyrimidine-conjugated AuNPs also induce drug resistance considerably slower than conventional, small-molecule antibiotics.

### 2.6. Silver NPs

Various properties, such as electrical, optical, physical, chemical, and thermal properties, influence the synthesis and utilities of metal-derived NPs for biomedical purposes. Some of these properties are important for medical applications, whereas others offer opportunities for industrial and environmental applications. The bactericidal characteristics of AgNPs are dramatically influenced by particle shape, size, concentration, and the colloidal state [49,50]. Smaller AgNPs sizes appear to increase biocompatibility and stability [51,52], for example, the antibacterial effects of AgNPs against *Staphylococcus aureus* and *Klebsiella pneumonia* were found to be enhanced when particle sizes of <30 nm were used [53]. In another study, 5–10 nm sized AgNPs exhibited bacteriostatic and bactericidal activity against *S. aureus*, methicillin-susceptible *S. aureus* (MSSA), and methicillin-resistant *S. aureus* (MRSA) [54]. It is worth noting that small-sized AgNPs interact with cell membranes; modify the lipid bilayer; increase membrane permeability; and finally, cause cell death [55]. Furthermore, AgNPs have been shown to interact with viruses, bacteria, and fungi in a particle shape-dependent manner [50,56,57,58], and 5–20 nm AgNPs were reported to hinder HIV-1 replication [59].

McShan et al. showed tetracycline-AgNPs and neomycin-AgNPs both acted synergistically to inhibit *Salmonella typhimurium* growth with half maximal inhibitory concentrations (IC_50_) of 0.07 μg/mL and 0.43 μg/mL, respectively [60]. AgNPs were also observed to dose-dependently reduce the viabilities of MRSA and non-MRSA; for this reason, neither are used in cultures at concentrations >1.35 × 10^−3^ μg/mL [61]. Lkhagvajav et al. compared colloidal AgNPs to AgNPs alone and found that they had greater antimicrobial activity in colloidal form [62]. Since AgNPs can only be applied as bactericidal agents in a liquid system on account of the low colloidal stability [63], the colloidal state of AgNPs is vital for the antibacterial activity. It offers strong antimicrobial therapy against infections, as it acts as a catalytic agent and destabilizes the enzymes that are specifically required for oxygen consumption by drug-resistant bacteria, yeast and fungi [59,63]. The enhanced bactericidal action of colloidal AgNPs has been confirmed against drug-resistant gram-positive and gram-negative bacteria and MRSA [62]. Zawadzka and coworkers prepared AgNPs on titania coatings and reported their excellent antimicrobial antibacterial stabilities and durabilities against *S. aureus*. Contact killing and released silver-mediated killing were hypothesized to be responsible for the bactericidal effects of these AgNP/TiO_2_ coatings [64].

Li and co-workers [65] investigated the antimicrobial activity and the mechanism of AgNP *E. coli* killing by studying the cell growths, membrane permeabilities, and morphologies of microbial cells. Growth assays revealed 10 μg/mL AgNPs completely inhibit the growth of 10^7^ colony-forming unit (cfu)/mL of *E. coli* cells. Permeability tests indicated an outflow of reducing sugars and proteins from bacterial cells, and the deactivations of respiratory chain dehydrogenases. These results show AgNPs have the potential to destroy the integrity of bacterial membranes. Treatment of *E. coli* cells with AgNPs at 50 μg/mL induced various morphological changes such as multiple pits and gaps in cell walls as confirmed by transmission and scanning electron microscopy. In addition, exposure to AgNPs at 10 μg/mL induced membrane vesicle solubilization and dispersal and the disorganization of membrane components. These findings suggest AgNPs cause bacterial cell membrane breakdown and enzyme deactivation in *E. coli* cells.

In an elaborate study on amoxicillin and AgNPs alone and in combination, the combination was found to reduce bacterial growth markedly more than that expected based on the results of single treatments [66]. Furthermore, the synergistic action of antibiotics with nanosilver for resistant and non-resistant bacteria differs. In resistant strains, differences in the mechanism of action between antibiotics and nanosilver are attributed to their enhanced activity. If a bacterial strain is resistant to one agent, another agent could kill the microbe in a different way, which could be understood more as an additive action rather than a synergistic effect. If there is no antimicrobial resistance in bacteria, the greater effect could be due to the binding reaction of amoxicillin and nanosilver. As regards their synergistic effects, antibacterials act by targeting points on cell surfaces, whereas AgNPs induce DNA damage (silver chelation of DNA prevents DNA unwinding). Durán et al. considered synergism between amoxicillin and AgNPs was due to the formation of sulfur bridges between amoxicillin molecules and AgNPs [67]. However, Li et al. suggested the hydrophobic nature of AgNPs enhances interactions with bacterial membranes, and thus, facilitates amoxicillin transport across cell membranes [66].

**Figure 3:**
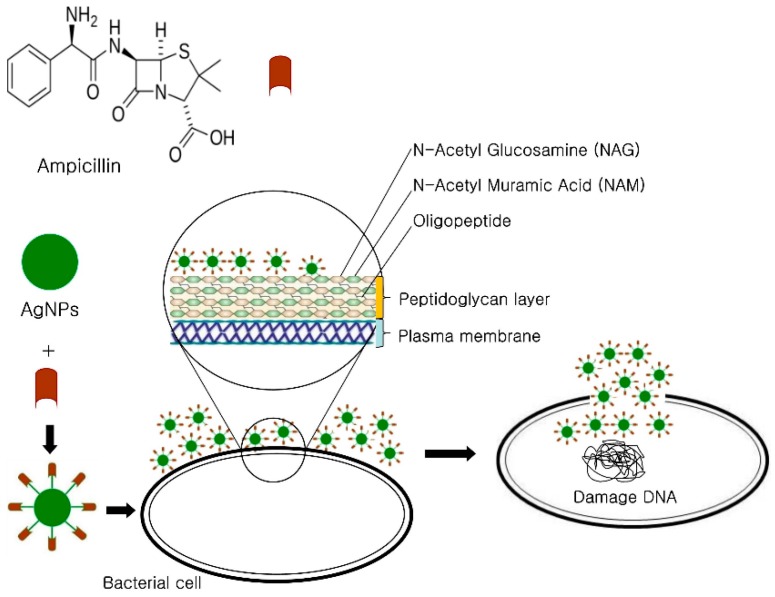
Proposed antibacterial mechanisms of ampicillin plus AgNPs. AgNPs-ampicillin in combination hinders the formation of crosslinks in the peptidoglycan layer, which results in cell lysis. AgNPs-ampicillin complex inhibits DNA unwinding.

Fayaz et al. synthesized polydispersed 5–40 nm AgNPs and confirmed their enhanced antibacterial activities against gram-positive and gram-negative bacteria when loaded with different antibiotics, and ampicillin was found to have the greatest effect. Furthermore, the formation of cross-links in the peptidoglycan layer was inhibited by ampicillin loaded AgNPs, which also hindered the unwinding of bacterial DNA (Figure 3) [68].

### 2.7. Zinc Oxide NPs

The mechanisms responsible for the antimicrobial activity of ZnO-NPs have yet to be clarified. Proposed mechanisms include (1) the destruction of cell integrity caused by direct contact between ZnO-NPs and cell walls [69], (2) ROS formation [70], and (3) the release of antimicrobial ions, mainly Zn^2+^ ions [69]. However, because the chemical nature of dissolved zinc depends on media constituents, it is likely that the mechanism of ZnO-NP toxicity is media dependent [69].

The photocatalytic efficiency of ZnO is high, and ZnO is more biocompatible than TiO_2_ [71]. Nirmala et al. [72] prepared ZnO-NPs using a DC thermal plasma and found in a photocatalytic study ZnO-NPs degraded methylene blue and ZnO-NPs inhibited bacterial growth (*Bacillus subtilis*, *E. coli*, *S. typhi*, and *S. aureus*). When exposed to UV radiation in aqueous solution, ZnO-NPs generate ROS, hydrogen peroxide (H_2_O_2_), and superoxide ions (O2^−^) [73,74], which can be exploited to target microbes. 

The toxic effect of ROS on bacteria is a result of their high reactivities and oxidizing properties [32]. Since aqueous ZnO-NPs suspensions produce significant amounts of ROS, ROS are regarded to be the main cause of nanotoxicity [75,76,77]. Photocatalytic ROS production is primarily responsible for the antimicrobial effects of several metal oxides [78]. Raghupathi and co-workers [79] suggested the strong antibacterial effect of ZnO was due to enhanced ROS generation in the presence of UV light. In addition, the toxicity of ROS is directly related to the damage caused to cellular components like lipids, nucleic acids, and proteins by the internalization of ROS. However, the relevance of ROS generation is debatable as some have observed ROS generation in the dark [80].

Navale and coworkers [81] studied the antimicrobial effects, growth inhibitions, and mechanistic events induced by synthesized ZnO-NPs in *S. aureus*, *S. typhimurium*, and *Aspergillus flavus* and *fumigatus*. A growth study revealed that ZnO-NPs (size 20–25 nm) had significant bactericidal effects on the fungi and bacteria studied, and an examination of the antibacterial effects of ZnO-NPs exposed to UV showed ZnO-NPs generated ROS. In addition, it was observed that the oxidation of NPs under γ-l-Glutamyl-l-cysteinyl-glycine induced oxidation stress was responsible for the antibacterial behavior of ZnO-NPs [81].

The antibacterial effects of these NPs were also studied by examining their inhibitory effects on the growth of *Campylobacter jejuni*, which is highly sensitive to ZnO-NPs. MICs of ZnO-NPs for *C. jejuni* ranged from 0.05 to 0.025 mg/mL, which were 8- to 16-fold lower than those for *Salmonella enterica* serotype Enteritidis and *E. coli* O157:H7, respectively, which were around 0.4 mg/mL. Furthermore, ZnO-NPs had bactericidal rather than bacteriostatic effects on *C. jejuni*. Scanning electron micrographs showed that the majority of *C. jejuni* cells transformed from spiral to coccoid morphologies after being exposed to 0.5 mg/mL of ZnO-NPs for 16 h, which concurred with morphological alterations observed under other stress conditions. Reverse transcription-quantitative PCR showed ZnO-NP exposure increased expression levels of *katA* and *ahpC* (oxidative stress genes) and *dnaK* (a general stress response gene) by 52-, 7-, and 17-fold, respectively, which suggested that oxidative stress and cell membrane disruption were responsible for ZnO-NP induced *C. jejuni* death [82].

**Figure 4:**
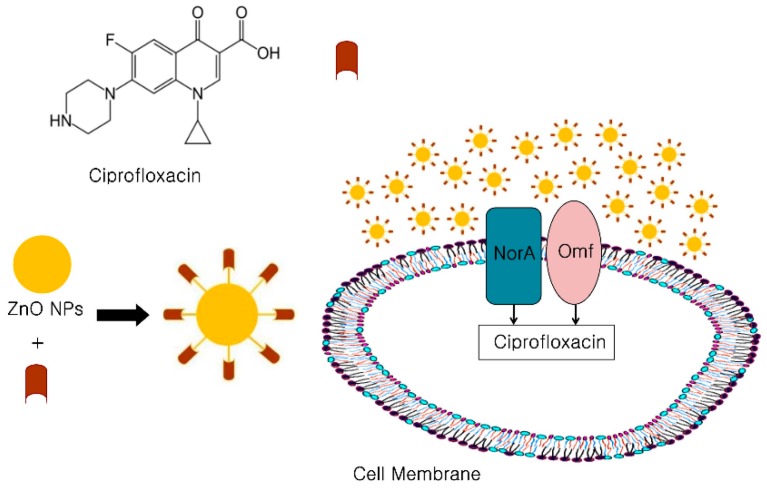
Schematic representation of the antibacterial mechanism of ciprofloxacin conjugated ZnO-NPs. ZnO-NP-Ciprofloxacin complex interferes with NorA (endogenous efflux transporter) and membrane Omf proteins, and thus enhances ciprofloxacin entry.

Ciprofloxacin with ZnO-NPs has also been reported to exhibit antibacterial activity against *S. aureus* and *E. coli*. Ciprofloxacin in the presence of ZnO-NPs increased zones of inhibition by 27% and 22% against tested strain of *S. aureus* and *E. coli*, respectively. Furthermore, ZnO-nanoparticles (500, 1000, and 2000 μg/disk) had a concentration-dependent effect on the antimicrobial potency of ciprofloxacin and enhanced the activity of ciprofloxacin against the tested strains. ZnO-NPs reportedly interfere with the pumping action of NorA protein, which facilitates active efflux and thus inhibits the antibacterial effect of ciprofloxacin. Another proposed activity involves the interactions of ZnO-NPs with the membrane Omf protein (associated with the quinolones permeation to cell membrane). Furthermore, these interactions were also found to enhance the penetration of ciprofloxacin to the cell interior (Figure 4) [83].

### 2.8. Titanium Dioxide NPs 

The antibacterial action of titanium dioxide (TiO_2_) NPs is photo-dependent [84,85,86] and attributed to free radical production. These free radicals affect bacterial LPS, peptidoglycans, and the phospholipid bilayer by causing peroxidation. A study that focused on the antimicrobial effects of TiO_2_ NPs and/or UV radiation established that their synergism significantly damaged the external membrane of *E. coli* [66]. TiO_2_ and UV radiation administered separately potently damaged the outermost LPS layer of *E. coli* cells, but not peptidoglycans, and caused cellular damage as evidenced twisting, rough appearances, and regular wrinkles resembling groove-like rifts. In addition, cells bulged at both ends and became wider and shorter, but generally retained their rod-like form. These alterations in bacterial morphologies indicated breakdown of the outermost cellular layer to some extent. In contrast, cells exposed to TiO_2_ and UV completely lost their typical rod shape and became elliptical and spheroplast-like. These observations suggest damage to the outer membrane, LPS, peptidoglycans, and the phospholipid layer. Interestingly, due to an intact inner membrane, bacterial cells were still alive and showed no evidence of lysis [86]. 

The combined activities of various antibiotics and TiO_2_ NPs (20 nm) against MRSA have also been studied [87]. Zones of inhibition were used to assess the interaction between NPs and antibiotics. In the presence of sub inhibitory concentrations of TiO_2_ NPs (10 μg/disk), the zone of inhibition was maximally enhanced around the disks with penicillin and amikacin (10 mm) followed by ampicillin > oxacillin > amoxicillin/cephalexin/cefotaxime/ceftazidime/vancomycin/streptomycin > erythromycin and clindamycin > tetracycline. Reasonable enhancement in the area of the zone of inhibition was characteristic for sulphazidime, ciprofloxacin, rifampicin, and cotrimoxazole (4 mm). These results show, TiO_2_ NPs significantly enhanced antibiotic efficacies against *S. aureus* when used in combination with antimicrobial agents [87]. However, the mechanism underlying this synergism between antimicrobial agents and TiO_2_ NPs remains to be determined.

### 2.9. Nitric Oxide Releasing NPs

Nitric oxide (NO) has a short half-life and is highly reactive, and its lipophilic nature means it can pass through cell walls. In addition, it can rapidly diffuse down concentration gradients from regions of NO production. The specificity of the effect of NO is dependent on the ability of nanoparticles to generate it at target sites at high enough rates to produce a concentration gradient [42]. The antimicrobial effects of NO are the subjects of current investigations.

NO-releasing silica NPs have been used as novel antimicrobials against *P. aeruginosa*. Co-condensation of tetra-alkoxysilane with amino-alkoxysilane modified with diazeniumdiolate NO donor was used to prepare NO-releasing NPs carrying large NO payloads. When the bactericidal efficacy of NO-releasing NPs was compared to 1-[2-(carboxylato) pyrrolidin-1-yl]diazen-1-ium-1,2-diolate (PROLI/NO; a small molecule NO donor), the NP-derived nitric oxide was found to have considerable more bactericidal efficacy. A cytotoxicity assay performed in an in vitro mammalian fibroblast system confirmed the non-toxic properties of NO-releasing silica NPs, whereas PROLI/NO showed significant toxicity to mouse fibroblasts cells subjected to bactericidal concentrations. These findings confirm the validity of NO-based strategies for combating bacterial infections [88]. Interestingly, Mihu et al. [89] showed that NO-NPs have therapeutic effects on *Acinetobacter baumannii* infected wounds, by demonstrating NO-NP treatment reduced microbial burden, suppurative inflammation, and collagen degradation.

Although NO is an important constituent of the host defense against invading pathogens, its therapeutic applications are limited due to the lack of a practical delivery system. In the recent past, a NO-releasing NP platform (NO-NP) exhibited wide-spectrum in vitro antibacterial activity and in vivo pre-clinical efficacy in a dermal abscess model. NO-NP-treated animals had fewer cfu/mg in infected tissue than control and vancomycin-treated animals. Furthermore, NO-treated animals showed less inflammatory infiltrate than controls and vancomycin animals. Notably, the direct bacterial killing and immunomodulatory properties of NO are superior to those of antibiotics, which normally act via a single mechanistic path, and for this reason, NO limits the risk of resistance development. It appears that NO-NPs offer a novel and straight forward means of treating deep tissue infections and abscesses either topically or by injection [90].

## 3. Adverse Effects of Nanomaterials

In addition to acting as antimicrobials, metal oxide NPs have innumerable applications in end uses as varied as medical diagnostics, therapeutics, sensors, cosmetics, solar cells, and coatings. AuNPs are considered relatively safe NPs, because particle cores are inert and non-toxic. In one study, spherical AuNPs (4, 12, or 18 nm) coated with different capping agents were found to enter leukemia cells but not to influence cellular functions [91]. On the other hand, it has been well established AgNPs can accumulate within the human body and in various organs, but especially in brain due to their ability to crossing the blood–brain barrier. AgNPs have also been identified in lungs, spleen, kidney, liver, and brain in exposed rats [92]. On the other hand, zinc-based nanomaterials have been reported to cause toxicity and membrane injury and to increase oxidative stress in mammalian cell lines [75]. TiO_2_, which is chemically inert, also has adverse effects in its nanoform, as it exhibits toxicity such as DNA damage, genetic toxicity, and lung inflammation [93]. 

In addition, many NPs are coated with flexible hydrophilic polymers, which usually contain polyethylene glycol, and thus these particles remain in the circulation for much longer times [94]. However, silica NPs have shown promising results and are relatively safe for oral administration [95]. Additionally, it has been demonstrated that NPs exert their antimicrobial activity by releasing heavy metals, which have oxidative stress-inducing effects in man, and thus can induce a wide range of physiological, biochemical, and behavioral dysfunctions [96]. More research is essential to further understand the toxic effects of metallic NPs. Thus, the therapeutic use of NPs still presents many challenges.

## 4. Conclusions and Future Prospectives

Despite the development of an array of methods to address microbial resistance, the increasing emergence of multidrug-resistant microorganisms emphasizes the need for new therapeutic options. The high biocidal activities of metallic NPs make them viable alternatives to antibiotics and promising antibacterial agents, but their toxic effects restrict current usage. However, studies of interactions between antibiotics and metallic NPs have focused on conventional antimicrobial agents. Metallic NP-based platforms are promising alone or in combination with antimicrobial agents and provide a possible means of overcoming drug resistance. Given their enormous therapeutic potential, understanding the modes of action responsible for the bactericidal properties of NPs becomes an imperative.

## Figures and Tables

**Table 1:** Types of nanomaterials and their possible antimicrobial mechanisms.

NP Types	Antimicrobial Mechanism	Citations
Gold	Heavy electrostatic attraction, accumulation at cell surfaces, and interaction with cell membrane	[37,38]
Silver	Interferes with cell membrane, damages DNA and electron transport	[39]
Zinc oxide	Disrupts the cell membrane, accumulates inside the cell and produces toxic H_2_O_2_	[40]
Titanium dioxide	Damages cell membranes and releases reactive oxygen species	[41]
Nitric oxide-releasing NPs	Releases nitric oxide and produces reactive oxygen species	[42]

## Abbreviations

NPs	Nanoparticles
ROS	Reactive Oxygen Species
LPS	Lipopolysaccharide
AuNPs	Gold nanoparticles
AgNPs	Silver NPs
MSSA	Methicillin-susceptible *Staphylococcus aureus*
MRSA	Methicillin-resistant *Staphylococcus aureus*
MIC	Minimum inhibitory concentration
ZnO-NPs	Zinc oxide nanoparticles
TiO_2_ NPs	Titanium dioxide nanoparticles
UV	Ultraviolet
NO-NPs	Nitric oxide nanoparticles
